# RGD-coated polymeric microbubbles promote ultrasound-mediated drug delivery in an inflamed endothelium-pericyte co-culture model of the blood-brain barrier

**DOI:** 10.1007/s13346-024-01561-6

**Published:** 2024-03-18

**Authors:** Christopher Hark, Junlin Chen, Julia Blöck, Eva Miriam Buhl, Harald Radermacher, Robert Pola, Michal Pechar, Tomáš Etrych, Quim Peña, Anne Rix, Natascha I. Drude, Fabian Kiessling, Twan Lammers, Jan-Niklas May

**Affiliations:** 1 Institute for Experimental Molecular Imaging (ExMI), RWTH Aachen University, Aachen, Germany; 2 Electron Microscopy Facility, Institute for Pathology, University Clinic RWTH Aachen, Aachen, Germany; 3 Institute of Macromolecular Chemistry, Czech Academy of Sciences, Prague, Czech Republic; 4 QUEST Center for Responsible Research, Berlin Institute of Health at Charité, Berlin, Germany

**Keywords:** Drug delivery, Sonopermeation, Ultrasound, Microbubbles, Blood-brain barrier

## Abstract

Drug delivery to central nervous pathologies is compromised by the blood-brain barrier (BBB). A clinically explored strategy to promote drug delivery across the BBB is sonopermeation, which relies on the combined use of ultrasound (US) and microbubbles (MB) to induce temporally and spatially controlled opening of the BBB. We developed an advanced in vitro BBB model to study the impact of sonopermeation on the delivery of the prototypic polymeric drug carrier pHPMA as a larger molecule and the small molecule antiviral drug ribavirin. This was done under standard and under inflammatory conditions, employing both untargeted and RGD peptide-coated MB. The BBB model is based on human cerebral capillary endothelial cells and human placental pericytes, which are co-cultivated in transwell inserts and which present with proper transendothelial electrical resistance (TEER). Sonopermeation induced a significant decrease in TEER values and facilitated the trans-BBB delivery of fluorescently labeled pHPMA (Atto488-pHPMA). To study drug delivery under inflamed endothelial conditions, which are typical for e.g. tumors, neurodegenerative diseases and CNS infections, tumor necrosis factor (TNF) was employed to induce inflammation in the BBB model. RGD-coated MB bound to and permeabilized the inflamed endothelium-pericyte co-culture model, and potently improved Atto488-pHPMA and ribavirin delivery. Taken together, our work combines in vitro BBB bioengineering with MB-mediated drug delivery enhancement, thereby providing a framework for future studies on optimization of US-mediated drug delivery to the brain.

**Graphical abstract:**

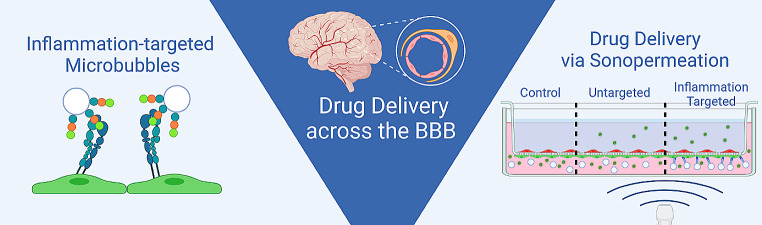

**Supplementary Information:**

The online version contains supplementary material available at 10.1007/s13346-024-01561-6.

## Introduction

The blood-brain barrier (BBB) serves as the interface between systemic circulation and the central nervous system (CNS). This barrier consists of a tightly controlled and highly coordinated network of different types of cells, including endothelial cells, pericytes, astrocytes, and neurons [1, 2]. Under physiological conditions, the BBB inhibits the passage of almost all systemically administered drug molecules into the CNS, allowing only highly lipophilic and relatively small molecule drugs to cross [3, 4]. Strategies to overcome the BBB have encompassed pharmacological approaches, like the use of ATP-binding-cassette-transporter blockers like quercetin or hyper osmolar agents like sorbitol, as well as physical interventions, such as intrathecal injection or sonopermeation [5–8].

Improved understanding of drug delivery to the brain profits from advanced in vitro setups, employing BBB models to study drug shuttling at the molecular level and upon different co-treatments [9–12]. A key parameter indicating proper BBB functionality is the transendothelial electrical resistance (TEER), which results from a confluent endothelial cell layer with intact tight junctions [13]. In mammals, TEER values are reported to exceed 1000 Ω⋅cm² in vivo. In vitro, values usually range between 40 and 800 Ω⋅cm², depending on the setup and cell lines used [14–16]. For the cells used in this study, comparably low TEER values from 10 to 50 Ω⋅cm² have been reported, which still allow measurements of TEER changes [17, 18].

A healthy, functional BBB carefully controls the transport of nutrients, hormones and molecules and restricts cellular passage in and out of the brain [19]. Upon inflammatory stress, endothelial cells react with modified expression of molecules, resulting in increased permeability, altered cellular structure, and/or signaling via receptors to mediate immune responses [20]. These changes induce invasion of immune cells across the BBB to combat disease progression and protect CNS tissue [21]. An example for these receptors is the group of integrins, that promote immune cell adhesion and interactions between cells and the extracellular matrix [22]. Among those, alpha-v-beta-3 integrin receptors can be exploited as a target for inflammation-targeted drug delivery systems, for example, via the Arg-Gly-Asp (RGD) peptide motif which is known to bind to several integrin subtypes [23, 24]. The stimulation of integrin expression can be induced in endothelial cells by exposure to e.g. tumor necrosis factor (TNF), of which the latter can be employed to establish an inflamed in vitro BBB model [25–27]. In contrast to animal models, in vitro models allow direct control over interacting cell types and stimuli [18, 28]. The use of in vitro models enables easier adjustment to research questions as well as high-throughput screening of neuro-active drugs and brain-targeted drug delivery systems, both alone and in combination with physical interventions intended to promote trans-BBB drug delivery, such as sonopermeation.

Sonopermeation is a biophysical mechanism based on the interaction of microbubbles (MB) with ultrasound (US) [29]. MB oscillate when exposed to acoustic pressures and can cavitate or burst depending on their shell type and applied US parameters [29, 30]. MB-mediated mechanical forces affect nearby cells, and can induce an opening of intercellular junctions and/or increased transcytosis [31]. The main effects of sonopermeation-mediated BBB-opening are observed in capillary beds, where the distance between MB and endothelial cells is small, and the thin vessel wall allows for easier molecular transport [32–34]. Here, sonopermeation can enhance tissue penetration of circulating small molecule drugs and larger compounds, e.g., antibodies and drug delivery systems, across the BBB [35, 36]. One concept to enhance the regional specificity of sonopermeation is the use of focused US (fUS), where an acoustic lens concentrates US waves in a small target volume. The efficacy of US-induced local BBB opening can be further increased by employing targeted MB, which are surface-coated with different ligands ranging from peptides to antibodies, that bind to and accumulate at specific sites of interest. Their mainly imaging-focused application is more and more extended to therapeutic applications [37, 38]. Here, their focused accumulation can be exploited to target different pathological lesions in the CNS for more effective sonopermeation-based BBB opening.

In vitro BBB models promote research on biophysical and pathophysiological mechanisms aiming for an improvement of, e.g., localized drug delivery. The increasing complexity of models can serve different research purposes, starting from monocultures up to 3D-bioprinted microfluidic models with incorporated flow or organoids [18, 39, 40]. While simpler models are used to screen for toxic effects or drug delivery mechanisms, more complex models can enhance understanding of cell-cell interactions of different cell types in a 3D setting. As an example, antitumor research has already employed in vitro BBB models incorporating glioma cell lines [28]. Apart from the general setup, the choice of cell lines heavily impacts the scope of application [1]. Both primary and immortalized rat or bovine cerebral endothelial cell lines provide high TEER values, around 600 Ω⋅cm², but they differ from human cells in terms of receptor expression [16]. For example, the expression of the solute carrier monocarboxylate transporter 1 is rarely reported in animal-based in vitro BBB models but is present in BBB models relying on human cell lines [16, 41]. Primary human stem cell-based BBB models can create a more realistic display of the human BBB, but their availability and handling tend to be challenging [42, 43]. Immortalized human cell lines can be a pragmatic compromise between decent TEER values, representative molecular marker expression, and ease of application [17]. BBB co-culture models incorporating such immortalized human cerebral cells can provide important new insights into the expression of potential target receptors, into the usefulness of drug delivery systems, as well as into the mechanisms of trans-BBB drug delivery.

We here describe the development of an in vitro co-culture BBB model based on human cell lines to study the application of sonopermeation for delivering a prototypic polymeric drug carrier system across a healthy as well as an inflamed BBB. To improve drug delivery at inflammatory sites, we employed RGD peptide-coated MB to increase the specificity of sonopermeation. The binding of RGD-coated MB to inflamed cerebral endothelium was evaluated under static and dynamic (flow) conditions, mimicking the in vivo situation. As a proof-of-concept for inflammation- and infection-targeted trans-BBB drug delivery, we finally also assessed the ability of sonopermeation to shuttle the antiviral small molecule drug ribavirin across the BBB.

## Materials and methods

### BBB model

**Fig. 1:**
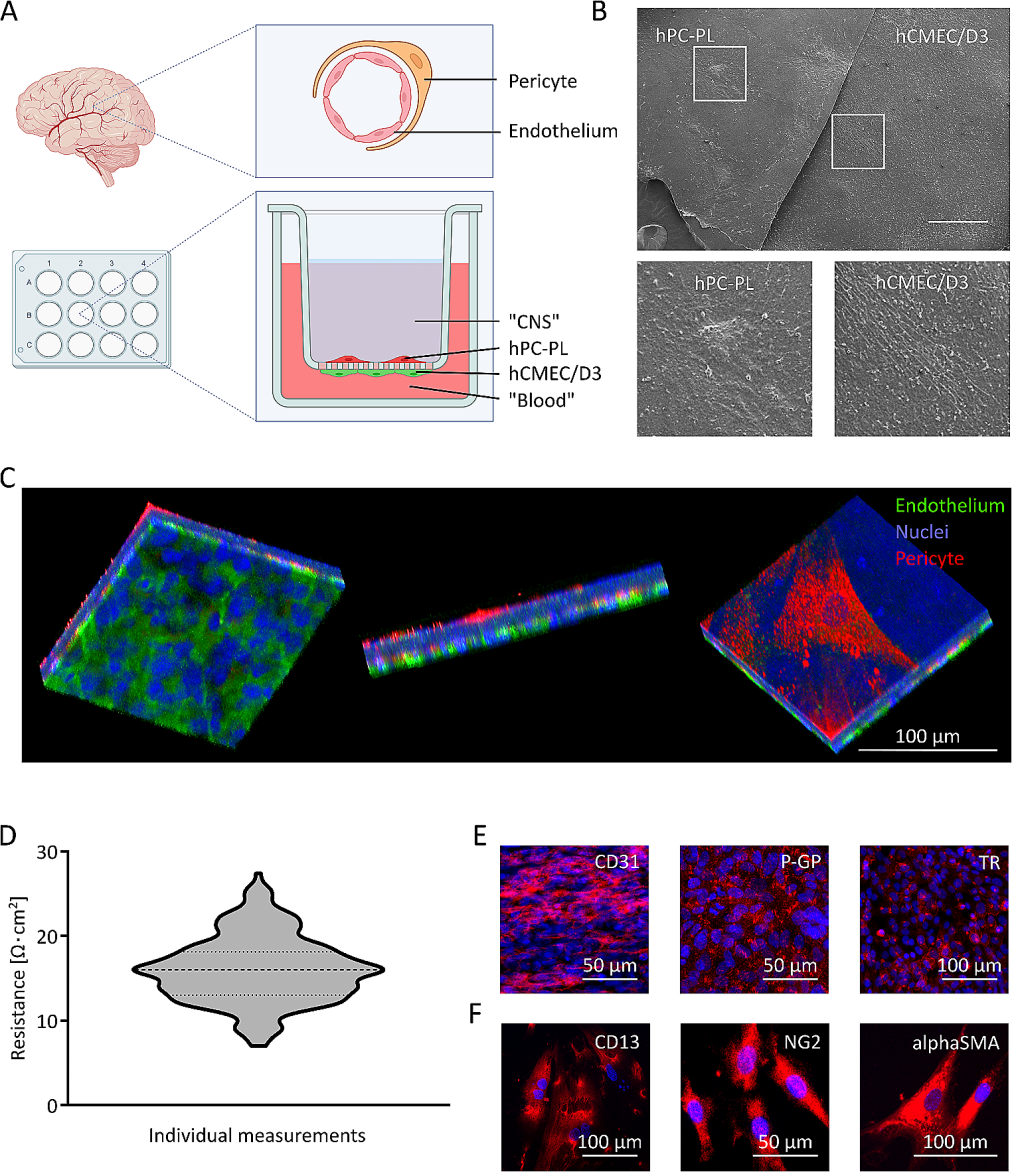
**In vitro BBB model.** A: Schematic depiction of an in vivo BBB capillary and of our in vitro BBB co-culture model. In this model, hCMEC/D3 endothelial cells and hPC-PL pericytes were used on opposing sides of a porous transwell membrane. hCMEC/D3 faced the model blood compartment, while hPC-PL faced the model CNS compartment. Graphic was created with Biorender.com B: Scanning electron microscopy image of the BBB model upon cutting and turning one part of the membrane, exposing scattered hPC-PL (left) on the upper side and a dense hCMEC/D3 layer at the bottom (right). Lower images are magnified from the overview image above, with white rectangles marking the areas in the overview image. Scale bar indicates 500 µm. C: Three-dimensional confocal microscopy of our BBB model, visualizing CD31-labeled endothelial cells in green and CD13-labeled pericytes in red. Nuclei in blue. Between the cell layers, blue fluorescence of the membrane can be observed. D: TEER values measured of in vitro BBB models. The central line indicates a median TEER value of 16 Ω⋅cm², the smaller dotted lines represent SD (n=264, including technical and biological replicates). E: Immunostainings (in red) of endothelial markers CD31, P-GP (P-glycoprotein) and TR (Transferrin receptor) by hCMEC/D3 cells in 2D cell culture. DAPI-stained nuclei in blue. F: Immunostainings (in red) of fibroblast markers CD13, NG2 (Neural/glialantigen 2), alpha-SMA (Alpha smooth muscle antigen) by hPC-PL cells. Nuclei in blue.

Human cerebral microvascular endothelial cells D3 (hCMEC/D3, Merck Millipore, Germany) were cultivated as advised by the manufacturer in EndoGRO™ medium (Merck Millipore, Germany) with 1 ng/mL FGF-2. Human pericytes (hPC-PL, PromoCell, Germany) were cultured according to the manufacturer´s instructions in Pericyte Growth Medium 2 (Promocell, Germany). For BBB models, 25,000 hCMEC/D3 cells were seeded on the lower side of a 6 mm polyester membrane cell culture insert with 0.4 μm pores (Corning® Transwell®, USA) and left to incubate and attach for 1 h. After placing the inserts into the well, 50,000 hPC-PL were seeded into the upper compartment. This generated a blood compartment on the endothelial side and a CNS compartment facing the pericytes (Fig. 1A). The model was left to establish for 15 days at 37°C with 5% CO_2_. Further experiments were conducted if stable and consistent resistance measurements indicated an intact barrier. To induce inflammation, the models were incubated with human TNFα (abcam, Great Britain) at a concentration of 20 ng/ml in medium for 4 h under standardized cell culture conditions [20].

### Synthesis of MB

Synthesis of poly-butyl cyanoacrylate (PBCA) MB was done using a previously established protocol [44]. In short, *n*-butyl cyanoacrylate was added to a 1% Triton X-100 solution and stirred to encourage bubble formation. MB were purified using repeated centrifugation and resuspension. Targeted MB were produced by coupling cyclo(RGDfK) (RGD-MB, Arg-Gly-Asp, MedChemExpress, USA) or unspecific control cyclo(RADfK) (RAD-MB, Arg-Ala-Asp, MedChemExpress, USA) amino acid sequences to the shell of hydrolyzed PBCA MB via carbodiimide conjugation, as described previously [45]. Labeling with rhodamine B was also done as previously described [30, 46, 47]. Unlabeled MB at a concentration of 1 × 10^9^ MB/ml were diluted in a 0.1 mg/ml rhodamine B solution and incubated for 4 h under continuous stirring. The solution was then cleared of broken MB and free rhodamine B via centrifugation and resuspension of the MB cake in fresh Triton X-100 solution until the solution below the cake was clear and colorless. Labeled MB were stored for up to 48 h at 5°C.

### Polymeric drug carrier

Atto488-labeled HPMA polymers (poly(*N*-(2-hydroxypropyl)methacrylamide; Atto488-pHPMA) were synthesized as described before [48, 49]. Briefly, *N*-(2-hydroxypropyl) methacrylamide (HPMA; 85 mol %) and 3-(*N*-methacryloyl glycylglycyl)thiazolidine-2-thione (Ma-GG-TT; 15 mol %) were combined in DMSO for radical copolymerization for 6 h at 50°C to produce the copolymer precursor poly(HPMA-co-Ma-GG-TT). Atto488-NH_2_ was added to the polymer precursor (10% w/w) in *N,N*-dimethylacetamide in the presence of *N,N’*-diisopropylethylamine in equimolar amount to Atto488. After 30 min, remaining polymeric reactive TT groups were aminolyzed with 1-aminopropan-2-ol, followed by a precipitation step with diethyl ether. Purification of the product was done by gel filtration on PD-10 desalting columns containing Sephadex G-25 resins in water. The resulting polymer had a molecular weight of 67 kDa and a polydispersity (Ð) value of 1.7. The dye content of Atto488 was 2.1% w/w as assessed by UV/Vis spectrophotometry. The polymeric nanocarrier size under physiological conditions was 10–20 nm, as measured via fluorescence correlation spectroscopy.

### TEER analysis

TEER measurements were performed using an EVOM2 epithelial voltohmmeter with STX-2 chopstick electrodes (World Precision Instruments, USA) at 37°C. The obtained values were normalized by subtracting the resistance of inserts without cells (44 ± 12 Ω⋅cm²) and relating them to membrane surface; thus, results are expressed in Ω⋅cm². A baseline TEER measurement of our BBB model was conducted after 15 days, as a quality control for barrier function. Longitudinal TEER measurements were performed in a water bath heated to 37°C. Models were returned to culture conditions between two measurements.

### Drug translocation

Model macro- and small molecule drugs were introduced to the blood compartment in the transwell BBB co-culture model at a concentration of 0.1 nmol/ml for Atto488-pHPMA or 100 µg/ml for ribavirin [50]. Drug concentrations in CNS compartments were determined at different time points using fluorescence detection or reverse-phase high-performance liquid chromatography (HPLC), respectively. For Atto488-pHPMA, fluorescence detection was done at an excitation wavelength of 490 nm. Emission was assessed at 525 nm using the Tecan Reader infinite M200. Sample preparation for HPLC analysis of ribavirin (Merck, Germany) was adapted from Homma et al. [51]. To measure drug concentrations, 150 µl of medium were extracted from the CNS compartment. Using 1 M HCl, the pH was adjusted to 2 to allow for protein precipitation. Samples were centrifuged at 5,000 g for 5 min, and 85 µl supernatant was taken for HPLC analysis with an isocratic elution method using methanol (containing 0.1% TFA) as a solvent in a 5 min sequence. A C18 column (4.6 × 150 mm, particle size 5 μm), 1 ml/min flow and a detection wavelength of 220 nm were used. The area under the curve (AUC) was correlated to a control sample and a previously done calibration curve to estimate the drug concentrations in µg/ml present in the sample.

### US and sonopermeation

The employed fUS setup consisted of a Siglent SDS1202X + oscilloscope (China), for monitoring signals as well as triggering a Keysight 33,600 A Series Trueform Waveform Generator (USA). The generated signal was intensified by an AG 1021 power amplifier (T&C Power Conversion, Inc., USA), stepped up in voltage and matched to the impedance of the Olympus V314-SU-F1.00IN-PTF US transducer (Germany), which was submerged in a de-ionized water bath preheated to 37°C. Inserts were placed in lumox® multiwell plates (Sarstedt, Germany) and fixed 2.5 cm above the transducer with the bottom of the plate still submerged. MB were diluted in 800 µl pre-warmed EndoGRO™ medium at a concentration of 1 × 10^7^ MB/ml. Untreated and TNF-treated BBB models were exposed to US with a frequency of 0.933 MHz, 10 cycles per pulse, a pulse repetition frequency (PRF) of 2 kHz, and a peak negative acoustic pressure of 200 kPa for 3 s. Additionally, BBB models were treated with US and either untargeted control MB or RGD-MB. For binding experiments, TNF pre-treated models were incubated in MB suspensions for 10 min at 37°C before submerging the models in fresh medium for the sonopermeation treatment. Afterwards, models were placed into new wells and subjected to further resistance measurements or translocation assays with Atto488-pHPMA or ribavirin. Models were returned to culture conditions between data acquisitions.

### Flow chamber experiments

300,000 hCMEC/D3 cells per 35 mm petri-dish were seeded and incubated under culture conditions for 6 days. Inflammation was induced using TNF as described above [20]. A custom-made flow chamber setup was used to evaluate the binding efficacy of RGD- and RAD-targeted PBCA MB to TNF-stimulated cells (Fig. 3B). Cells were pre-labeled with Hoechst (1:500, nuclei) and WGA-488 (1:500, ThermoFisher Scientific, USA, cell membranes) for 30 min. Cells were exposed to rhodamine B-labeled RGD-MB or RAD-MB in EndoGRO™ medium at 1 × 10^7^ MB/ml with a flow of 2 mm/s, to be comparable to in vivo physiology [52–54]. The time point of analysis was immediately after exposure. For RGD-MB + TNF, additional observation time points were set after 10 or 30 min of exposure to medium at a flow of 2 mm/s. Unbound bubbles were removed by washing with medium and PBS (1 min medium, 1 min PBS, 1 min PBS). The area of cells exposed to the flow was immediately investigated using fluorescence microscopy via an Axio Imager M2 fluorescence microscope (Carl Zeiss, Germany). For every petri-dish, five images were acquired using the 10x objective. Bound MB were counted manually and unbound MB, defined by not being in observable contact to cell membranes, moving bubbles and/or signals out of focus plane, were excluded from the final data (NB: on average less than one MB per picture).

### Microscopy

BBB models were fixated in 3% glutaraldehyde in PBS, dehydrated with ethanol and coated with a 10 nm layer gold/palladium for scanning electron microscopy (SEM, ESEM Quattro S, ThermoScientific FEI, Netherlands). For immunostainings, a fixation with 4% paraformaldehyde buffered in PBS for 12 min was performed. These models were then incubated with primary antibodies to detect the expression of the following targets: P-glycoprotein (P-GP, 1:100, ab170904, abcam, Great Britain), transferrin receptor (1:200, ab84036, abcam, Great Britain), alpha-v-beta-3 integrin (1:250, ab7166, abcam, Great Britain), CD13 (1:250, ab108310, abcam, Great Britain) and CD31 (1:200, RA0259-C.5, ScyTek Laboratories, USA). Incubations with primary antibodies were done overnight at 4°C, followed by a washing step with PBS to remove unbound antibodies before incubation with secondary antibodies for 1 h at room temperature (1:500, 711-166-152, Dianova, Germany and 1:150, 115-165-166, Dianova, Germany). For tight junction staining, fixation was done with ice-cold methanol and acetone 1:1 for 10 min at -20°C [55]. The ZO-1 antibody (1:100, ab190085, abcam, Great Britain) was left on the fixated models at 4°C overnight, washed with PBS and visualized via a secondary antibody exposure for 1 h at room temperature (1:500, 711-166-152, Dianova, Germany). In all stained models, nuclei were counter-stained using DAPI (1 µg/ml), while WGA-488 (1:500, ThermoFisher Scientific, USA) was used to identify cell membranes. Models were incubated with both agents at room temperature for 10 min. A minimum of three fluorescence microscopy images per staining were acquired using an Axio Imager M2 fluorescence microscope (Carl Zeiss, Germany). Confocal microscopy was performed using an inverted Zeiss LSM 710 confocal laser scanning microscope running under ZEN black 2.3 SP1 FP3 software (version 14.0.27.201) using a 20x/0.8 Plan-Apochromat objective (Zeiss, Oberkochen, Germany). 405 nm, 488 and 561 nm laser lines were used for the excitation of DAPI, Alexa 488 and Cy3, respectively. According to the combination of dyes, beam splitters and filters were chosen as suggested by the Smart Setup function of the ZEN software to achieve optimal separation of the signals.

### Statistical analysis

Data are presented as the mean ± standard deviation (unless specified otherwise). Statistical analysis was performed using GraphPad Prism 10 (GraphPad Software, San Diego, CA, USA). Analysis was done using ANOVA with Tukey´s post-hoc test. P values < 0.05 were considered statistically significant. Significant differences are indicated as * = *p* < 0.05, ** = *p* < 0.01, *** = *p* < 0.001, **** = *p* < 0.0001.

## Results

### BBB co-culture model generation and characterization

The BBB model was generated by co-culturing hCMEC/D3 endothelial cells and hPC-PL pericytes on opposing sides of a porous membrane (Fig. 1A). SEM imaging of mature models showed a dense layer of endothelial cells on the lower side of the membrane with pericytes scattered across the upper side. The low pericyte coverage accurately reflects the in vivo situation where not every part of a brain capillary is covered by pericytes [56]. Both sides were visualized within one image by cutting the membrane in half and turning one part around so that hPC-PL and hCMEC/D3 were simultaneously visible (Fig. 1B). Cell identities were confirmed using immunostainings for the differentiation markers platelet endothelial cell adhesion molecule (CD31, endothelial cells) and alanine aminopeptidase (CD13, pericytes), depicting clear separation of the cell types without cross-contamination (Fig. 1C) [57, 58]. TEER values of our BBB model were in the range of 16 ± 4 Ω⋅cm² (*n* = 264, Fig. 1D) and dropped to values of a membrane without cells after EDTA stimulation (Fig. S1). Immunostainings revealed the expression of characteristic cellular molecules for endothelial cells and pericytes. Endothelial hCMEC/D3 expressed CD31, efflux pump P-glycoprotein (P-GP) and the transferrin receptor (TR). hPC-PL presented CD13, neural/glial antigen 2 (NG2) and alpha-smooth muscle antigen (alphaSMA) (Fig. 1E, F). This, in combination with sufficient cell coverage and appropriate TEER values, showed that our BBB model was viable and suitable to proceed to drug delivery experiments.

### Opening of the in vitro BBB with sonopermeation

**Fig. 2:**
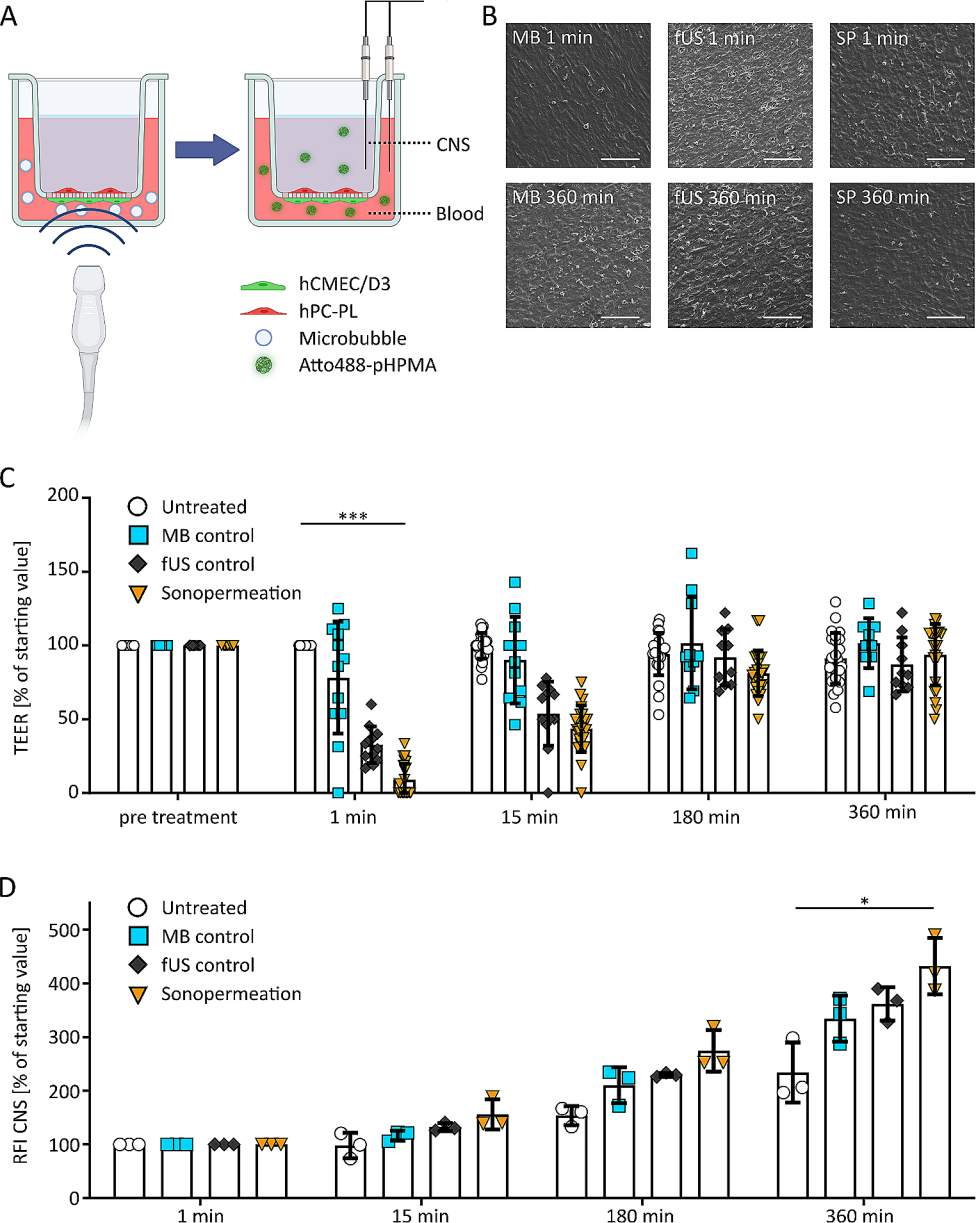
**Sonopermeation in the in vitro BBB model.** A: Illustration showing sonopermeation application in the BBB co-culture model, followed by TEER measurements and Atto488-pHPMA nanocarrier translocation from the blood to the CNS compartment. Graphic was created with Biorender.com. B: Scanning electron microscopy images of the BBB model at different time points after exposure to MB, US application (fUS) and sonopermeation (SP), respectively. No differences were observed between the treatments, indicating that no major damage was done to the BBB by SP treatment. Scale bar is 100 µm. C: TEER in the BBB co-culture model upon sonopermeation vs. control conditions. *** indicates p<0.001. n≥12per time point. D: Translocation of Atto488-pHPMA nanocarrier across the model BBB into the CNS compartment, determined by the relative fluorescence intensity (RFI). After 360 min, a significant difference of SP treatment to all control groups was observed. * indicates p<0.05 (n=3 per time point).

Our BBB model was exposed to fUS, MB and a combination of both to evaluate the effect of sonopermeation on TEER and nanocarrier translocation (Fig. 2A). While the untreated BBB model showed stable TEER values, sonopermeation at 0.933 MHz, pulse repetition frequency 2 kHz and 200 kPa decreased TEER to approx. 10% of baseline values (Fig. 2C). The overall model integrity after sonopermeation treatment was confirmed using stainings for nuclei and cell membranes, as well as SEM imaging (Fig. 2B). We were unable to detect cell detachment or visible holes in the endothelial cell layer in randomly chosen samples after treatments (Fig. S2). TEER values gradually returned to starting values over a time window of 6 h, indicating that the treatment was well-tolerated, and that BBB opening was reversible (Fig. 2C). Cells treated only with MB without sonication presented with a negligible decrease in TEER. Only fUS without MB also decreased TEER values in our setup, but significantly less than the combination of MB and US (Fig. 2C). When evaluating Atto488-pHPMA translocation, there were no significant differences between MB-only treatment and US-only treatment (Fig. 2D). Conversely, beginning 15 min after sonopermeation, while not statistically significant, there is an increase of signal in the CNS compartment for the macromolecule, pointing to a greater translocation, which is significantly enhanced over time ((*p* < 0.05); Fig. 2D).

### Targeting inflammation under capillary flow conditions

**Fig. 3:**
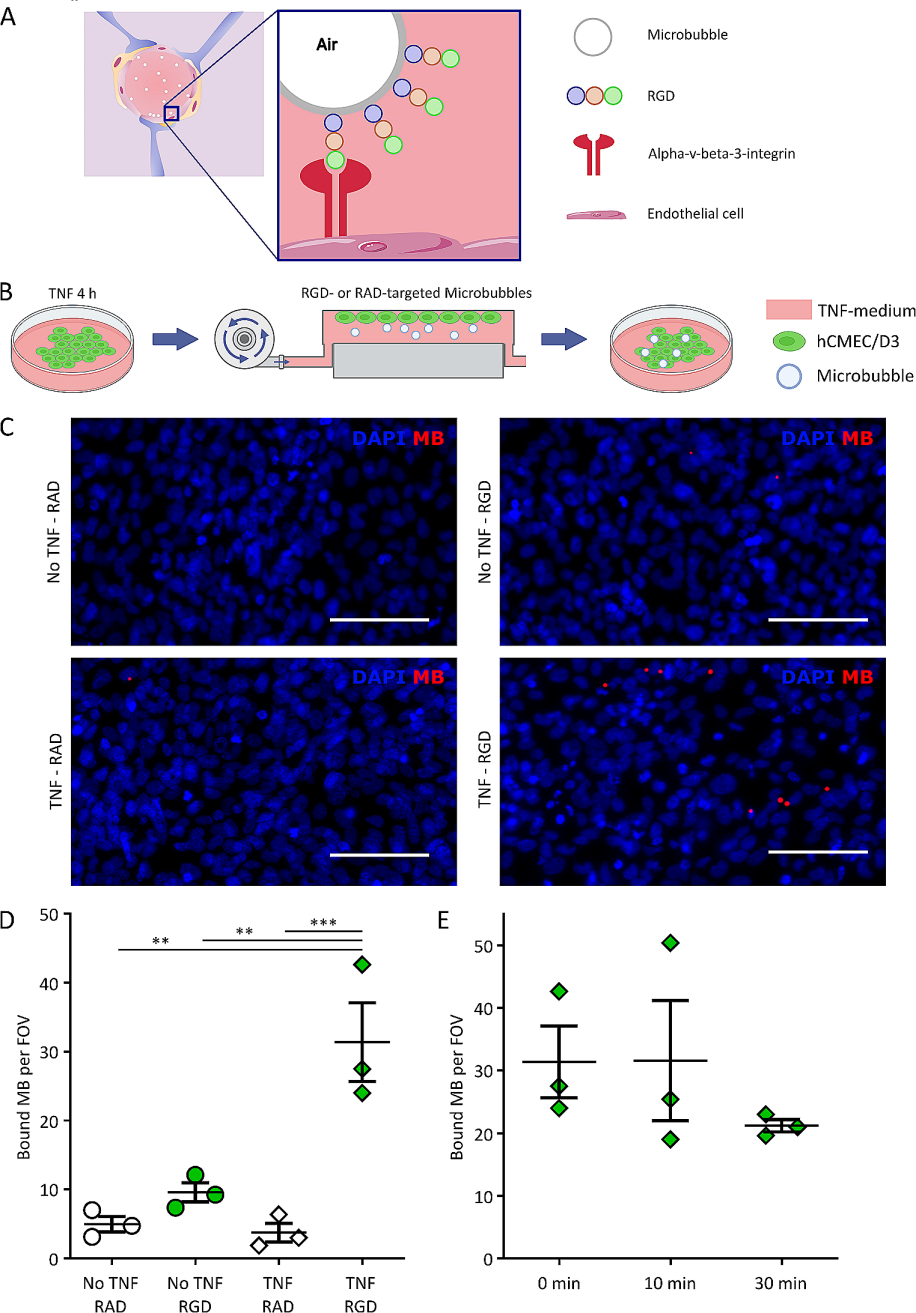
**MB-binding to inflamed BBB under flow conditions.** A: Illustrationdisplaying the interaction between RGD-MB and integrin-expressing endothelial cells in case of an inflamed BBB. B: Schematic showing of the flow chamber MB binding experiments. First, endothelial cells were activated with TNF to induce inflammation. The cell layer was exposed to different labelled MB formulations at capillary flow speeds for 10 min. EM: endothelial medium, RAD: RAD-MB, RGD: RGD-MB. Graphic was created with Biorender.com. C: Fluorescence microscopy analysis revealed specific binding of labeled RGD-coated (but not RAD-coated) MB to inflamed brain endothelial cells. FOV: Field of view. Scale bar represents 100 µm. D: Bound MB were quantified per field of view after 10 min of exposure at capillary flow (Mean ± SEM of n=8 replicates). ** indicates p<0.01, *** indicates p<0.001. E: MB bound to inflamed endothelium were exposed to capillary flow speeds for 0, 10, or 30 min (Mean ± SEM of n=8 replicates).

Inflammatory sites in the body are known to express different molecular markers such as alpha-v-beta-3-integrin, which is induced in vitro by, e.g., the application of TNF. Endothelial cells (hCMEC/D3) were exposed to TNF after 5 days of growth. The targeting behavior of RGD-MB and RAD-MB as a negative control to inflamed and untreated hCMEC/D3 was evaluated at capillary flow, allowing the binding of MB to expressed integrins. After a washing sequence, a mean value of 1 MB per image taken was observed to be defined as unbound due to no contact to cell membranes, movement and/or signals outside the focus (Fig. 3A, B). Compared to RGD-MB at unstimulated endothelium and RAD-MB under both conditions, a significant increase of bound RGD-MB was seen on inflamed endothelial layers at a flow speed of 2 mm/s (Mean ± SEM: 5 ± 2, 10 ± 2, 4 ± 2, 31 ± 10 bound MB for No TNF + RAD, No TNF + RGD, TNF + RAD and TNF + RGD respectively, Fig. 3C, D). Exposing the TNF + RGD condition to fresh, MB-free medium at 2 mm/s for up to 30 min demonstrated a long-term binding of MB (32 ± 17, 21 ± 2 bound MB upon 10 and 30 min exposure respectively, Fig. 3E).

### Sonopermeation via targeted MB at the inflamed BBB

**Fig. 4:**
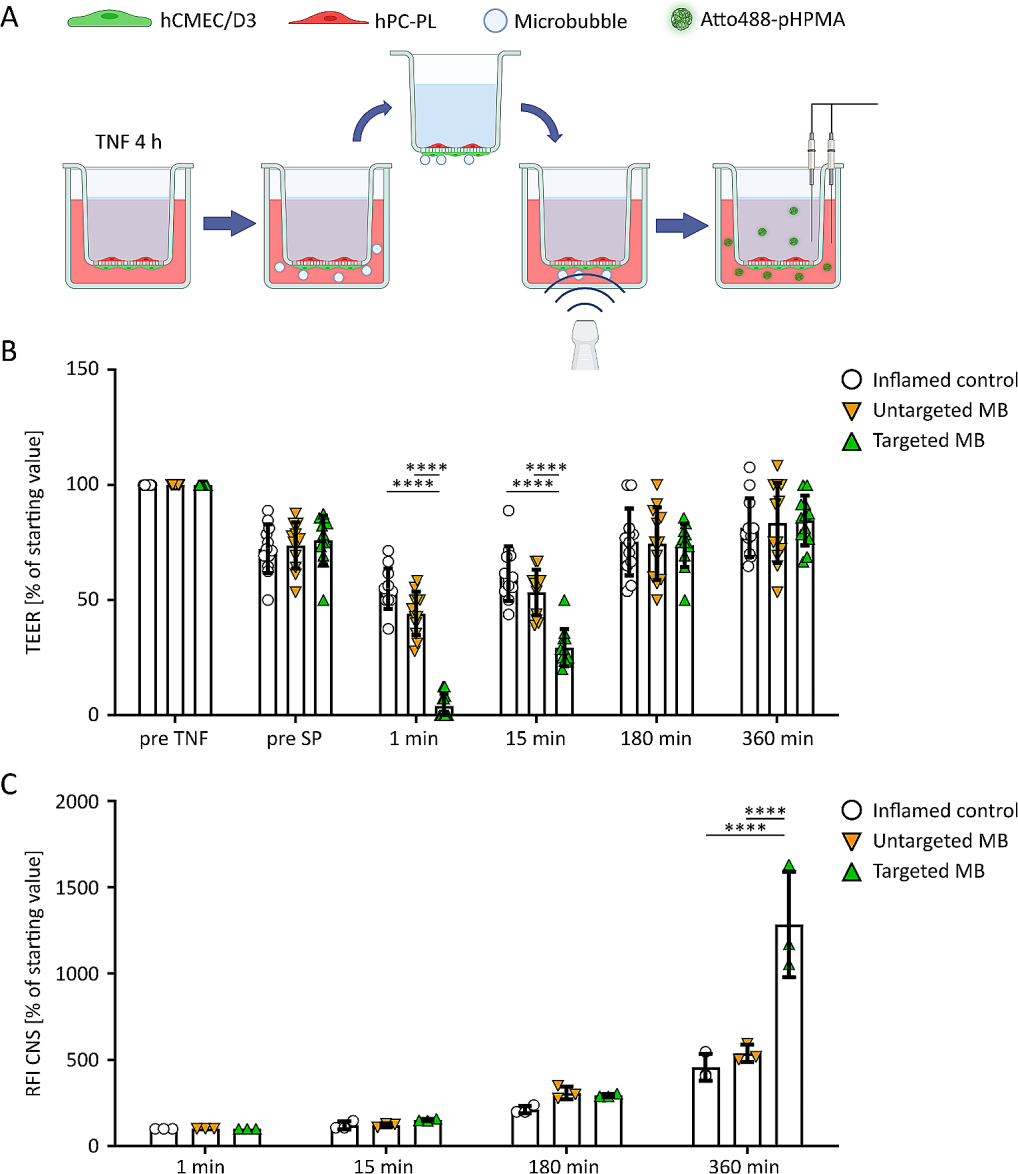
**RGD-MB induced sonopermeation in the inflamed BBB model.** A: Schematic overview of the experimental workflow. Graphic was created with Biorender.com. B: TEER values in the inflamed BBB model incubated without(control), with untargeted and with RGD-targeted MB up to 360 min post fUS treatment. Sonopermeation (SP) with RGD-MB potently reduced electrical resistance in the inflamed BBB models. Over time, a gradual recovery of TEER values was observed. No significant difference between control (no MB) and untargeted MB was detected, indicating complete removal of unbound MB during transwell transfer. **** indicates p<0.0001. n=12 per time point. C: Translocation of Atto488-pHPMA nanocarrier across the inflamed BBB co-culture model upon sonopermeation was assessed by analysis of relative fluorescence intensity (RFI) in the CNS compartment. The combination of US and RGD-MB resulted in an increase in RFI. **** indicates p<0.0001; n=3 per time point.

In vivo, unbound MB are cleared from systemic circulation relatively rapidly, typically within a timeframe of minutes. We mimicked this situation in our in vitro BBB model via incubation with untargeted and RGD-targeted MB for 10 min, followed by a transfer of the transwell insert into a new well, ensuring the removal of unbound MB (Fig. 4A). When comparing TEER values at different time points after US application, only RGD-MB were found to be able to reduce TEER, to 4 ± 5% of starting values (Fig. 4B). This was followed by a recovery of the TEER values over time. For untargeted MB, no differences in TEER modulation versus control conditions were observed (Fig. 4B). In line with the TEER values, Atto488-pHPMA translocation to the CNS compartment of the model was significantly improved only after sonopermeation treatment with RGD-MB (Fig. 4C). Interestingly, this was only observed at 360 min post treatment. The difference in timing, with TEER values dropping immediately after sonopermeation versus nanocarrier translocation only happening at relatively late time points after sonopermeation can be explained by the slower diffusion of larger molecules such as our nanocarrier when compared to the ion transit measured by TEER. Additionally, transcytosis-mediated translocation most likely plays a role in facilitating nanocarrier translocation at later time points after sonopermeation.

**Fig. 5:**
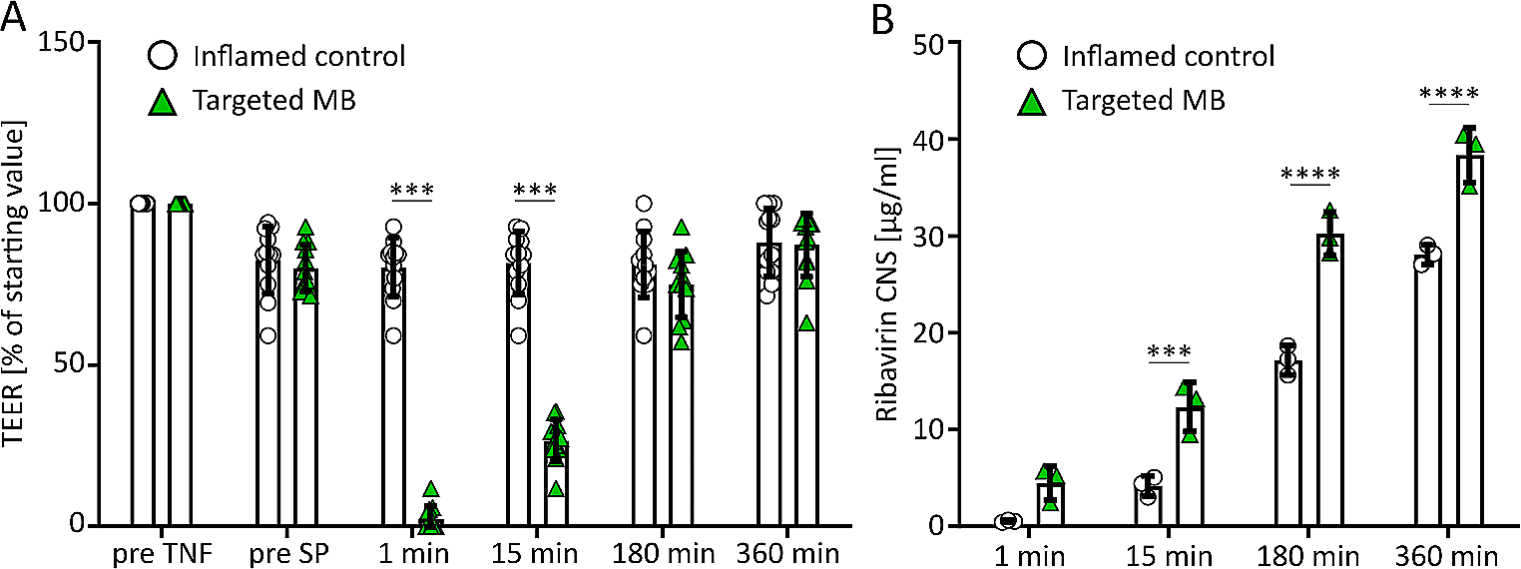
**Sonopermeation with RGD-MB increased ribavirin translocation across the inflamed BBB.** A: TEER values in the TNF-treated BBB co-culture model upon treatment with targeted MB and sonopermeation (SP) over time up to 360 min post application. After a strong initial decline in resistance, a gradual recovery to control values was observed. *** indicates p<0.001; n=12 per time point. B: Translocationof ribavirin across the BBB model was analyzed by determining drug concentration in the CNS compartment using HPLC. *** indicates p<0.001, **** = p<0.0001, n=3 per time point.

After confirming the efficacy of RGD-targeted MB-mediated sonopermeation with the fluorophore-labeled polymeric nanocarrier, we employed the inflamed BBB model in combination with targeted MB to assess the effect of sonopermeation on the delivery of the clinically approved small molecule antiviral drug ribavirin. Ribavirin is incapable of passing the BBB under physiological conditions due to its hydrophilicity but could potentially be used against various viral CNS infections [59]. As shown in Fig. 5A, TEER resistance upon sonopermeation significantly dropped to 2 ± 4% of starting values, in line with the results presented in Fig. 4B. TEER values also again recovered to those of controls after less than 6 h. Ribavirin concentrations in the CNS compartment were already significantly enhanced 15 min after sonopermeation and remained higher than those of controls at all following time points (Fig. 5B). The differences in timing for pHPMA nanocarriers and ribavirin were expected due to size and polarity differences. While pHPMA is a weakly hydrophilic compound and a comparably large molecule, the hydrophilic and small ribavirin can translocate across the BBB model much faster, provided that openings through sonopermeation are present.

## Discussion

In this study, we demonstrated the successful application of an in vitro BBB model based on immortalized human cell lines to study sonopermeation-mediated delivery of a nanosized drug carrier and a clinically approved small molecule drug under different (patho)physiological conditions. Our results show that (1) a healthy and inflamed BBB model with decent TEER values can be generated; (2) sonopermeation with both targeted and untargeted MB enhances delivery across the BBB; (3) both nanocarriers and free drugs can cross the BBB in our model.

The BBB co-culture model has been established by us and coworkers previously, and it is adapted here for sonopermeation research [28]. For this specific purpose, the cell orientation was changed to move the endothelial cells to the lower side of the membrane, enabling close interaction between MB and this cell layer (Fig. 1A,C). In this configuration, both cell lines expressed markers typical for BBB cells (Fig. 1E,F) [2, 60]. The model provided reliable TEER values of around 16 Ω⋅cm², which is in range with published TEER values for hCMEC/D3 cells (i.e. 10 to 50 Ω⋅cm² [17, 18]), indicating a functional BBB required for drug delivery studies (Fig. 1D). EDTA, a chelating agent which removes Ca^2+^ from tight junctions and thereby inhibits tight junction function, served as a positive control for BBB opening, and - when applied to the endothelial side - resulted in a fast drop of TEER (Fig. S1) [61]. Sonopermeation with fUS and PBCA MB was also able to reversibly open the in vitro BBB, improving the passage of Atto488-pHPMA (Fig. 2B-D) [35]. The observed recovery time of our BBB model after opening is comparable to observations made in animal studies [62]. In humans, closure of the BBB in less than 1 day has been observed when appropriate fUS parameters were chosen [63, 64]. Increased delivery of Atto488-pHPMA was seen immediately after sonopermeation treatment but only became statistically significant at 6 h after sonopermeation. This can likely be explained by the slow diffusion of relatively large molecules like 10–20 nm-sized pHPMA polymers across opened junctions, as well as by increased transcytosis (i.e. a slow process) induced by the oscillating MB during sonopermeation [29]. To dissect possible contributions of transcytosis to the observed, improved drug delivery, studies including transcytosis blockers or lower operating temperatures would be possible. Modifying such parameters also influences other factors like the known interaction between temperature and TEER and merits further studies in the future.

By introducing TNF in our BBB model, we established a setup to study inflammatory reactions of the BBB in vitro. We confirmed stable and specific binding of RGD-coated MB to alpha-v-beta-3 integrins, which are upregulated on endothelial cells in inflamed tissues and in response to TNF, at a flow speed comparable to murine and human brain capillaries (Fig. 3D,E) [52, 54]. Previous studies have shown rapid clearance of non-targeted MB from the bloodstream within less than 10 min [47]. The observed stable binding of RGD-coated MB to inflamed BBB endothelium over 30 min allows specific targeting of inflamed vessels, paralleled by removal of unbound MB from the bloodstream, thereby avoiding sonopermeation effects on uninflamed blood vessels within the sonicated area (Fig. 3E) [65]. This is a promising finding for further studies, although we expect faster clearance of bound MB in vivo, as the fluid dynamics of blood with possible contact of bloodborne cells and MB differ from our experimental settings in vitro. We emulated the removal of unbound MB from the bloodstream in our setup by switching the MB-exposed BBB models into fresh medium before fUS treatment, and demonstrated efficient BBB opening by targeted sonopermeation, which resulted in increased delivery of both Atto488-pHPMA nanocarrier and the small molecule drug ribavirin (Figs. 4 and 5). The effect of trans-BBB translocation was much faster for ribavirin than for Atto488-pHPMA. This is not surprising, given the significantly smaller size of ribavirin (< 1 nm) vs. pHPMA (10–20 nm), which contributes to much more rapid diffusion across the opened BBB, and also to less dependence on e.g. transcytosis. Improved targeted delivery of the clinically used ribavirin under inflammatory conditions encourages further research combining sonopermeation-induced BBB opening with anti-inflammatory drug delivery. Studies on the BBB involvement in CNS diseases linked to neuroinflammation such as tumors, encephalitis and autoimmune diseases could provide insights into new treatment options by opening the BBB for drugs previously incapable of reaching the CNS.

Due to the simplification of a complex three-dimensional anatomical structure in living organisms to a simplified two-dimensional cell co-culture model, the results reported here serve as a starting point for future (US-mediated) trans-BBB delivery research. Our BBB model provides a balance between resembling a human BBB while keeping required resources (and ethical/animal aspects) manageable [18]. More sophisticated in vitro models may provide insights into the interplay of additional cell types or stimuli [16], while less complex models, containing for instance only endothelial cells, may be more suitable for high-throughput studies. When considering sonopermeation, US settings that have been identified in vitro have to be adjusted for a loss of pressure due to tissue attenuation in vivo. There have been different approaches to limit this loss in animals and patients by overcoming the skull, either implanting the transducer intracranially or employing advanced extracranial transducer setups [63, 64, 66]. In this context, one has to keep in mind that our BBB model serves - as all in vitro or in vivo models; with their individual advantages and disadvantages– primarily a tool to promote very basic to early preclinical stage research on the ability of different US and MB combinations to help shuttle drugs and drug delivery systems across a model BBB, and that it enables large-scale screening studies and mechanistic analyses that would not be possible in in vivo setups.

## Electronic supplementary material

Below is the link to the electronic supplementary material.

**Supplementary Material 1. Figure S1:** EDTA treatment of mature BBB models. Electrical resistance values measured of in vitro BBB models before and after 5 min exposure to 0.5 mmol/l EDTA at 37.5°C. A mean TEER value of 14 Ω · cm1 dropped to 3 Ω · cm2 after exposure. Data was analyzed using an unpaired t-test. **** indicates p>0.0001; n=4.

**Supplementary Material 2. Figure S2**: SEM images of treated BBB models. Scanning electron microscopy images of the BBB model at different time points after exposure to MB, US application (fUS) and sonopermeation (SP), respectively. No differences were observed between the treatments, supporting that no major damage was done to the BBB by SP treatment. Scale bar is 100 μm.

## Data Availability

The materials used and datasets generated are available from the corresponding authors upon reasonable request.
