# Supplementary figures and images for: RGD-coated polymeric microbubbles promote ultrasound-mediated drug delivery in an inflamed endothelium-pericyte co-culture model of the blood-brain barrier
Source: Drug Deliv Transl Res. 2024 Mar 18;14(10):2629–41. doi: 10.1007/s13346-024-01561-6 (PMC11383844; doi:10.1007/s13346-024-01561-6)

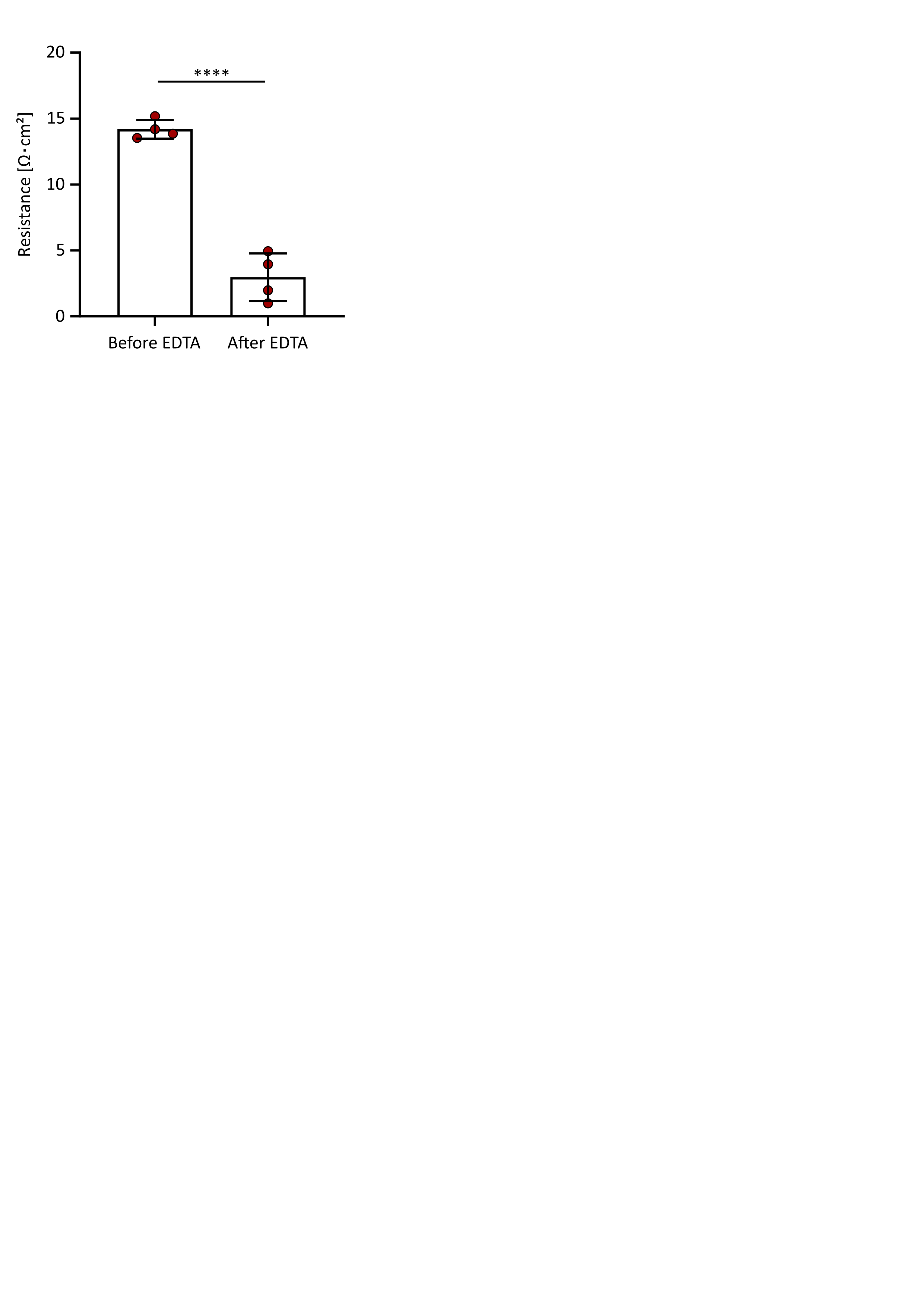

Supplement: Supplementary file 1 — Supplementary Material 1. Figure S1: EDTA treatment of mature BBB models. Electrical resistance values measured of in vitro BBB models before and after 5 min exposure to 0.5 mmol/l EDTA at 37.5°C. A mean TEER value of 14 Ω · cm1 dropped to 3 Ω · cm2 after exposure. Data was analyzed using an unpaired t-test. **** indicates p>0.0001; n=4. [file 13346_2024_1561_MOESM1_ESM.tiff]

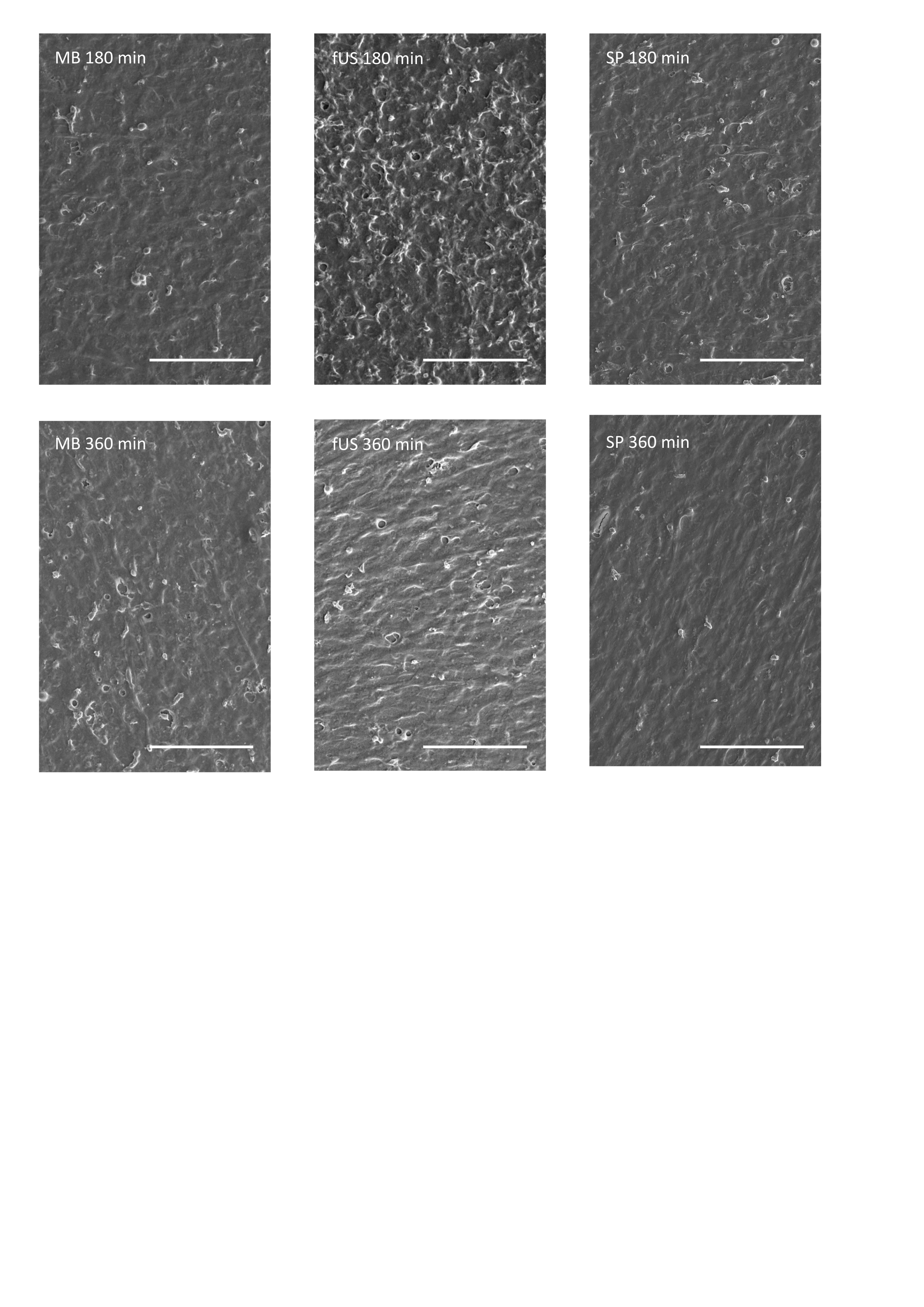

Supplement: Supplementary file 2 — Supplementary Material 2. Figure S2: SEM images of treated BBB models. Scanning electron microscopy images of the BBB model at different time points after exposure to MB, US application (fUS) and sonopermeation (SP), respectively. No differences were observed between the treatments, supporting that no major damage was done to the BBB by SP treatment. Scale bar is 100 μm. [file 13346_2024_1561_MOESM2_ESM.tiff]
